# Visit of French Doctors

**Published:** 1908-06-27

**Authors:** Leonard Williams

**Affiliations:** 8 York Street, Portman Square, W.


### EDITOR'S LETTER-BOX.

### VISIT OF FRENCH DOCTORS.

Sir,—As you are already aware, a party of French doctors is coming over on July 12 for a week. They will be accompanied by Professor Landouzy, the "Doyen de le Faculté." It has been arranged that the Doyen shall give a lecture—in his own language, of course—at 20 Hanover Square, at 5 p.m. on Tuesday, July 14. I am very anxious to get him a good audience, and I feel sure that you will assist me to do so. He is the best speaker amongst the many wonderful orators which they have amongst the professors, and it would be worth anybody's while to go and hear him. The subject will be "Some French Health Resorts."

Yours faithfully,
Leonard Williams.

8 York Street, Portman Square, W., June 22, 1908.

